# From Structure to Circuits: The Contribution of MEG Connectivity Studies to Functional Neurosurgery

**DOI:** 10.3389/fnana.2016.00067

**Published:** 2016-06-21

**Authors:** Elizabeth W. Pang, O. C. Snead III

**Affiliations:** ^1^Division of Neurology, Hospital for Sick ChildrenToronto, ON, Canada; ^2^Neurosciences and Mental Health, SickKids Research InstituteToronto, ON, Canada; ^3^Department of Paediatrics, Faculty of Medicine, University of TorontoToronto, ON, Canada

**Keywords:** magnetoencephalography (MEG), connectivity, epilepsy surgery, intractable epilepsy, functional mapping

## Abstract

New advances in structural neuroimaging have revealed the intricate and extensive connections within the brain, data which have informed a number of ambitious projects such as the mapping of the human connectome. Elucidation of the structural connections of the brain, at both the macro and micro levels, promises new perspectives on brain structure and function that could translate into improved outcomes in functional neurosurgery. The understanding of neuronal structural connectivity afforded by these data now offers a vista on the brain, in both healthy and diseased states, that could not be seen with traditional neuroimaging. Concurrent with these developments in structural imaging, a complementary modality called magnetoencephalography (MEG) has been garnering great attention because it too holds promise for being able to shed light on the intricacies of functional brain connectivity. MEG is based upon the elemental principle of physics that an electrical current generates a magnetic field. Hence, MEG uses highly sensitive biomagnetometers to measure extracranial magnetic fields produced by intracellular neuronal currents. Put simply then, MEG is a measure of neurophysiological activity, which captures the magnetic fields generated by synchronized intraneuronal electrical activity. As such, MEG recordings offer exquisite resolution in the time and oscillatory domain and, as well, when co-registered with magnetic resonance imaging (MRI), offer excellent resolution in the spatial domain. Recent advances in MEG computational and graph theoretical methods have led to studies of connectivity in the time-frequency domain. As such, MEG can elucidate a neurophysiological-based functional circuitry that may enhance what is seen with MRI connectivity studies. In particular, MEG may offer additional insight not possible by MRI when used to study complex eloquent function, where the precise timing and coordination of brain areas is critical. This article will review the traditional use of MEG for functional neurosurgery, describe recent advances in MEG connectivity analyses, and consider the additional benefits that could be gained with the inclusion of MEG connectivity studies. Since MEG has been most widely applied to the study of epilepsy, we will frame this article within the context of epilepsy surgery and functional neurosurgery for epilepsy.

## Introduction

With the advent of high resolution non-invasive neuroimaging, there has been improved ability to map the structure of the brain, as well as its connections. The idea that brain connections can be diagrammed as a “connectome” (Salvador et al., 2005; Sporns et al., 2005; Achard et al., 2006) has generated great hope that this “connectome” will provide the framework whereby we can understand the neural basis of human function and disease. The result was a significant paradigm shift in the field of neuroscience from a primarily modular, segregated view of brain function to a new view of integration amongst brain regions. According to this latter connectionist perspective, brain function depends on a network of widely distributed, interconnected circuits that communicate between distant brain regions to integrate incoming information and produce a coordinated output.

Understanding brain connectomics included investigations into both structural and functional connectivity, as these concepts are both interdependent and complementary. There is increasing evidence that brain areas communicate both along established physical pathways as well as functional pathways that may not be directly tied to brain structure, and thus the study of one is integral to the study of the other. In terms of structural connectivity, magnetic resonance imaging (MRI) has elucidated the anatomical arrangement of brain regions while diffusion tensor imaging (DTI) protocols in tandem with tractography algorithms, has supplied information on structural connections by tracking the course of myelinated fibers that travel between distant brain regions (Clayden, 2013).

The first methods for measuring brain functional connectivity were achieved using resting state functional MRI (fMRI) scans which identified pairs of brain regions showing time-correlated fluctuations in blood oxygen levels (Biswal et al., 1995). There are now a large number of studies which attest to the value of resting state fMRI for examining functional connections between brain regions (for reviews, see van den Heuvel and Hulshoff Pol, 2010; Van Essen, 2013). These investigations of functional connectivity are key as it is thought that it is the functional connections in the brain that are the drivers of cognitive behaviors.

Magnetoencephalography (MEG) is another imaging modality able to track functional connectivity (for a review on MEG, see Hari and Salmelin, 2012). MEG single-channel systems were developed in the 1970s with the first commercial whole-head MEG systems becoming available in the 1990s. It was only in the early 2000s that MEG use gained traction and attention with more standardized scanning and analysis protocols (Stufflebeam, 2011; Hari and Salmelin, 2012). The technique of MEG is based upon the elemental principle of physics that an electrical current generates a magnetic field. Hence, MEG uses highly sensitive biomagnetometers to measure extracranial magnetic fields produced by intracellular neuronal currents. Source localization of epileptic spikes and evoked responses as determined by MEG are co-registered with MRI and termed magnetic source imaging (MSI). MEG is primarily sensitive to signals arising from regions where the apical dendrites are tangentially oriented to the skull and scalp surface. Unlike electroencephalography (EEG), MEG signals are unaffected by tissue defects of the brain and skull bone, and unlike fMRI, abnormal hemodynamics in vascular malformations do not distort the MEG signal. So, MEG is a neurophysiological modality which measures the summed neuronal activity of small cortical patches covering several millimetres with a time scale in the milliseconds. Localization of this activity allows mapping of brain regions involved in specific functions. However, the high sampling rate of MEG recordings also allows for measurement of a wide bandwidth of oscillatory responses, and the computation of the correlated (by phase and/or amplitude) oscillatory activity between distinct cortical patches also gives a metric of functional connectivity. Thus, like fMRI, MEG can offer both localization and connectivity information.

Table 1 provides a comparison between the neuroimaging modalities discussed thus far. As can be seen, each neuroimaging modality presents with its own set of advantages and disadvantages, and in all likelihood, it will be the convergence of these methods that will generate the next big leaps of knowledge in the field. As fMRI is a more mature technology, much has been written about it; however, MEG, as a younger technology, is not as well known but holds promise. We think it important that clinician neuroscientists, and not just basic neuroimaginers, understand the potential that MEG has to address some of the gaps in our understanding of neural circuitry and functional connectivity. Further, while using a segregationist model, MEG has become an established clinical tool for localizing epilepsy foci and basic functions. With the paradigm shift to a network perspective of the brain, we think there is room for developing studies of MEG functional connectivity to assess whether MEG can accurately identify functional networks, and whether the applications of MEG functional connectivity studies can be expanded into other domains, including neurosurgery.

**Table 1:** **Comparison of non-invasive neuroimaging methods for acquiring connectome data**.

Modality and substrate	Advantage	Disadvantage		
MRI: anatomy	• High resolution images of brain structure	• Only provides anatomical information
DTI: water diffusion	• High resolution images of fiber tracts	• Only provides information on structural connectivity
fMRI: blood oxygen consumption	• High spatial resolution • Able to localize function • Measures resting state and task-based functional connectivity	• Relatively slower timing resolution • Only measures lower frequency oscillations
MEG: synchronous firing of neuronal populations	• Millisecond temporal resolution • Broadband measurement of spectral data • Relatively good spatial localization when used with a good source reconstruction algorithm • Measures resting state and task-based functional connectivity	• Uncertain as to sensitivity for *n* = 1 measurements and clinical decisions

In summary, the high resolution of MEG in the spatial, temporal and spectral/oscillatory domains allows tracking of synchronous neuronal firing (i.e., measuring evoked responses) and computations of oscillatory connectivity (i.e., measuring induced or intrinsic networks across a wide bandwidth of frequencies) as it relates to functions and behaviors. Further, advances in neurochemistry link the substrates of neurotransmission with the neuronal oscillatory activity such that a comprehensive picture of brain function, from chemistry to neuronal activity to blood flow and metabolism, is emerging. These represent the additional important contributions that MEG can offer to our understanding of cognition-related brain dynamics.

The information gained from MEG connectivity studies is synergistic with, and complementary to, the information gained from MR connectivity studies (Brookes et al., 2011), and together, these can offer new perspectives on brain function and structure. The paradigm shift in neuroscience, which conceives of the brain as holistic and integrated (as opposed to functionally segregated as will be described below), raises questions both as to the impact of neurosurgery on the function of the whole brain circuit and the ability of the rest of the circuitry to re-shape itself to compensate and maintain function in the face of brain injury and disease. These questions are important to the neurosurgical community because the answers invite a broader perspective on brain organization of function which can translate into new thinking about functional neurosurgery and possibly improved outcomes. MEG is a tool which is directly relevant to exploring and addressing these questions.

In this article, we begin by describing the traditional use of MEG for functional neurosurgery, particularly within the context of pre-surgical mapping for epilepsy surgery. We continue with a discussion of recent advances in MEG connectivity analyses and its application to the identification of both functional and dysfunctional networks. We end with our assessment of these MEG methods and present our thoughts on future applications of these methods to functional neurosurgery.

## MEG for Pre-Surgical Functional Mapping: The Localization Model

Traditional neurosurgery has relied on the visual identification of neuroanatomical landmarks. In fact, neurosurgery as a specialty has grown from increasing empirical support for the idea that specific brain functions were linked to discrete areas of the cortex (Penfield and Jasper, 1954). With contributions from histological, lesional and functional studies, the primary sensory cortices were located: to the striate cortex for visual functions, Heschl’s gyrus for auditory functions, and the post- and pre-central gyri for sensorimotor functions (for a review, see Mesulam, 1998). Using post-mortem and lesion studies, early clinicians elucidated specific left hemisphere brain centers allocated to speech production and comprehension; the first higher-order cognitive function to be localized (for a historical overview, see de Almeida et al., 2014). These findings supported a localization model of function.

As stated above, MEG sensors capture magnetic signals generated by the synchronous firing of a group of neurons. These data are visualized, like an EEG, as a plot of time on the *x*-axis against the size of the magnetic field strength on the *y*-axis. Source reconstruction algorithms have been developed that can compute the location of the brain source(s) that could have generated the observed signal. By asking the participant to complete a task, it is expected that segments of the recording corresponding to task processing would show regional increases in magnetic activity relative to a “baseline” or a time period with no task activity. Identification of the sites showing increased magnetic field strength (that is, neuronal activity) above baseline is thought to “localize” the generators involved in producing the function of interest. This computational process is referred to as “solving the inverse problem”, and this solution can be applied at every active time point so that a spatiotemporal activation profile is created (for a review, see Simos et al., 2000). While there are several valid approaches to solving the inverse problem, each has its own set of limitations and as long as users are aware of the unique limitations of their selected algorithm and interpret their results with these limitations in mind, any source reconstruction algorithm could be used (Stufflebeam, 2011).

The use of MEG to localize basic sensory functions is well established (for a review, see Stufflebeam et al., 2009) and a set of clinical guidelines (Burgess et al., 2011) are available that outline the minimum standards required for routine clinical recordings of somatosensory, basic hand motor, auditory and visual function. The first studies to map the somatosensory cortex using somatosensory evoked fields (SEF) in the MEG were conducted in control adults and recorded median and ulnar nerve stimulations, as well as stimulation of the individual digits. The results showed an orderly somatotopic representation on the posterior bank of the central fissure, and it was concluded that MEG recordings had sufficient sensitivity to resolve finger and hand topography in the somatosensory region of the brain (Baumgartner et al., 1991). These methods were applied to identify the central sulcus pre-surgically, and confirmed with direct cortical mapping (Gallen et al., 1993), as well as visual inspection of MR images (Sobel et al., 1993). In one of the first reports using pre-surgical SEF recordings in adult patients with drug-resistant frontal lobe partial seizures, the authors demonstrated high accuracy for localization of the post-central gyrus and central sulcus (Smith et al., 1994), a finding later confirmed in children (Minassian et al., 1999). In fact, in cases of cortical dysplasia, where cerebral function may not be in expected anatomical areas, MEG has demonstrated utility for identifying somatosensory cortices even when they are located outside the Rolandic areas (Burneo et al., 2004). The propensity of sensorimotor cortices to functionally reorganize makes it imperative to identify the central sulcus and map sensory and motor cortical regions (Nakasato and Yoshimoto, 2000).

In tandem with studies designed to map the somatosensory cortex, groups interested in motor function have used voluntary finger and hand movements in the MEG to identify the location of the pre-central gyrus (Weinberg et al., 1990; Kristeva et al., 1991; Nagamine et al., 1994; Pang et al., 2008). The accuracy of these methods is greatly improved with the application of electromyography electrodes on the limb of interest, as this allows precise identification of movement onset (Gaetz et al., 2009; Pang et al., 2009). However, while voluntary finger and hand movements can be easily completed by cooperative adults, repetitive voluntary movements are challenging in young children, and in clinical conditions where there may be involuntary movements, movement disorders, or reduced motor control (e.g., stroke, epilepsy). Thus, there is interest in finding alternative ways to activate the motor cortex, for example using motor imagery (Burianová et al., 2013; Kraeutner et al., 2014) or passive movements (e.g., Onishi et al., 2013). These methods are still under development but hold promise for their potential utility in patients unable to comply with the requirements for completing repetitive voluntary movements.

Like the SEF, both the auditory evoked fields (AEF) and visual evoked fields (VEF) are straight forward and easy to map. Using tone stimulation, the primary auditory cortex on the superior temporal gyrus is identifiable (Papanicolaou et al., 1990) and has been found to have a tonotopic organization (Pantev et al., 1995). Further, there is evidence that epilepsy in the primary auditory cortex impairs auditory processing ability in adults (Kubota et al., 2007) and children (Korostenskaja et al., 2010); however, it is only in extreme cases, such as deafness where there is dramatic functional reorganization of auditory areas (for a review, see Gordon et al., 2011). Using binocular (Harding et al., 1994) and monocular (Seki et al., 1996) pattern-reversal stimulation, the topographic organization of the primary visual cortex within the calcarine fissure can be identified. In cases where there is loss of visual function, as in patients with a partial or homonymous hemianopsia, the unaffected function continues to show reliable localization and excellent correlation with the cortical anatomy (Nakasato et al., 1996), and again, there is not substantial reorganization of visual cortices except in cases of a complete loss of function as in blindness (for a review, see Kupers and Ptito, 2014).

In summary, for identification of primary sensory areas around major anatomical landmarks such as the central sulcus, superior temporal gyrus or calcarine fissure, visual identification during functional neurosurgery may be sufficient. However, in cases where there is concern about cortical re-organization, MEG is invaluable because it is an easy, non-invasive method to quickly confirm the adequacy of the localization model although other non-invasive functional mapping methods (for example, fMRI) would also suffice for basic sensory mapping.

## When Localization Models are Insufficient: Mapping Language in the Brain

The localization model of language has been the backbone of language research for decades. A framework for basic language function was built upon lesion studies whereby injuries to specific brain areas produced stereotyped deficits in the affected patients. Those data suggested that in a healthy adult, language is subsumed in the left hemisphere within two perisylvian regions. The anterior region, also known as Broca’s area, is located in the pars opercularis and pars triangularis of the inferior frontal gyrus while the posterior region, known as Wernicke’s area, is located in the posterior portion of the superior temporal gyrus and adjacent parietal cortex. At its most basic level, the former region is thought to be involved in the production of speech while the latter is involved in the comprehension of language (Geschwind, 1970).

The advent of fMRI allowed intact persons to have their language tested non-invasively, and the first reports were that the Broca-Wernicke model mostly held true (Binder et al., 1995). While language was dominant in the left hemisphere, the borders of the classic Broca and Wernicke Areas were broader than originally thought, and there was greater intermixing of function and less sharp distinctions between expressive and receptive language (Binder et al., 1997; Binder, 1997).

Early MEG studies centered around receptive language function and it was demonstrated that by presenting words in the auditory modality, posterior language areas could be activated, and a laterality index calculated to determine the language dominant hemisphere (Breier et al., 2000). This calculation of language dominance showed high concordance with the intracarotid amobarbital procedure in children (Breier et al., 2001) and adults with epilepsy (Papanicolaou et al., 2004), as well as with fMRI (Billingsley-Marshall et al., 2007). More recently, protocols were developed to enable localization of frontal cortical areas involved in language production (Herdman et al., 2007) and validated against fMRI (Pang et al., 2011). Most recently, methods have been developed for assessing receptive language dominance under sedation (Rezaie et al., 2014) or sleep (Van Poppel et al., 2012). Because these new advances are in the research domain and still require extensive validation, the current clinical MEG guidelines recommend using language studies to index language laterality, but do not give definitive recommendations for methods to localize language (Burgess et al., 2011).

As more language neuroimaging studies are conducted, it has become increasingly apparent that there is a higher prevalence of atypical language lateralization and localization in patients with a neurological condition. For example, patients with mesial temporal lobe epilepsies (Pataraia et al., 2004), complex partial seizures (Breier et al., 2005), medically refractory epilepsy (Kadis et al., 2007) or stroke (Breier et al., 2004) were often found to show atypical language representation. Further, an initial case study (Kamada et al., 2006), followed by a set of case series (Gage et al., 2011; Eliashiv et al., 2014), reported a dissociation of language dominance. In all of these MEG studies, patients with left temporal lobe epilepsy showed receptive language dominance shifted to the right hemisphere while expressive language dominance was lateralized to the left. As well, studies of language lateralization using MEG (Kadis et al., 2011) and fMRI (Holland et al., 2007) in the typically developing brain showed bilateral and diffuse language representation in the young brain that became more left lateralized with age and expertise. Clearly, the left hemisphere frontal-temporal perisylvian model of language localization is inadequate in patients with disease and in developing children, and neurosurgery based solely on visual identification of language-related neuroanatomical landmarks would place the patient at increased risk for incurring language deficits post-operatively.

## From Segregation to Integration: Mapping Brain Functional Networks

The major shortcoming of the localization model is the premise that function is subsumed in an anatomically distinct region of the cerebral cortex, without consideration of the connections between and within regions. For example, we know that Broca’s area is connected to Wernicke’s area via the arcuate fasciculus, we know there are also connection to primary sensory and secondary association areas, and we know that these areas communicate and feedback to each other; however, localization models using a segregation approach cannot account for this complexity. Recent advances in computational neuroscience and improvements in computational processing power, have made it possible to augment studies of structural connectivity, which are anchored in physical anatomical pathways, to include measures of functional and effective connectivity, which describe network communication and integration (for a review, see Friston, 2011).

Therefore, in order to gain a fuller understanding of brain function and dynamics, there has been a shift towards connectionist paradigms which provide metrics to describe the level of communication and integration between distinct brain areas. Further, this shift has been driven by new understanding that the resting brain is not truly “at rest” (for a review, see Raichle, 2015). The traditional experimental approaches using brain evoked responses treated the non-task-related activity as “noise”, which required time-locked averaging to enhance the signal and decrease the noise. However, it is now known that there are active functional networks when the brain is “at rest”, and these involve not just the default mode network, but intrinsic networks for sensory, motor, language and attentional functions (e.g., Lowe et al., 1998; Cordes et al., 2001; Seeley et al., 2007). The elucidation of the relation between small cell assemblies containing as few as 600–800 cortical neurons and their relation to activation patterns in fMRI as seen by the blood-oxygen level (BOLD) contrast (Logothetis et al., 2001; Logothetis and Pfeuffer, 2004), led to later findings that, in fact, network communication was driven by neuronal oscillatory signals (Ikegaya et al., 2004). Thus, we have seen a convergence whereby we increasingly understand the relation between the hemodynamic signal, intrinsic neuronal oscillations and basic neurophysiological function (Lu et al., 2007; He et al., 2008; Khader et al., 2008).

## Definitions and Computations: Functional and Effective Connectivity

There is excellent body of foundational work describing the initial investigations into fMRI and MEG functional connectivity. Table 2 provides a list of the seminal works in this field. As well, to aid the interested reader, we include also a list of recommended articles that give either a comprehensive overview or present thoughtful critiques on this topic. The following paragraphs are intended to give a brief summary of basic concepts and introduce frequently used terminology.

**Table 2:** **Recommended list of (a) seminal papers and (b) reviews and commentaries on functional connectivity, in chronological order**.

**(a) Seminal papers: modality, authors, title**
MRI	Friston (1994)	Functional and effective connectivity in neuroimaging: a synthesis
	Bullmore et al. (1996)	Functional magnetic resonance image analysis of a large-scale neurocognitive network
	Biswal et al. (1997)	Simultaneous assessment of flow and BOLD signals in resting state functional connectivity maps
	Büchel and Friston (2001)	Interactions among neuronal systems assessed with functional neuroimaging
	Koch et al. (2002)	An investigation of functional and anatomical connectivity using magnetic resonance imaging
	Greicius et al. (2003)	Functional connectivity in the resting brain: a network analysis of the default mode hypothesis
	Salvador et al. (2005)	Neurophysiological architecture of functional magnetic resonance images of human brain
MEG	Gross et al. (2001)	Dynamic imaging of coherent sources: studying neural interactions in the human brain
	Stam (2004)	Functional connectivity patterns of human magnetoencephalographic recordings: a “small-world” network?
	Schnitzler and Gross (2005)	Functional connectivity analysis in magnetoencephalography
**(b) Reviews and commentaries: authors, title**
Horwitz (2003)	The elusive concept of brain connectivity.
Sporns et al. (2005)	The human connectome: a structural description of the human brain.
Friston (2005)	Models of brain function in neuroimaging.
Bassett and Bullmore (2006)	Small-world brain networks
Hagmann et al. (2010)	MR Conectomics: principles and challenges
Yeo et al. (2011)	The organization of the human cerebral cortex estimated by functional connectivity.
He et al. (2011)	Electrophysiological imaging of brain activity and connectivity—challenges and opportunities.
Toga et al. (2012)	Mapping the human connectome.
van Diessen et al. (2015)	Opportunities and methodological challenges in EEG and MEG resting state functional brain network research.

*Full citations are contained in reference list*.

**Figure 1:**
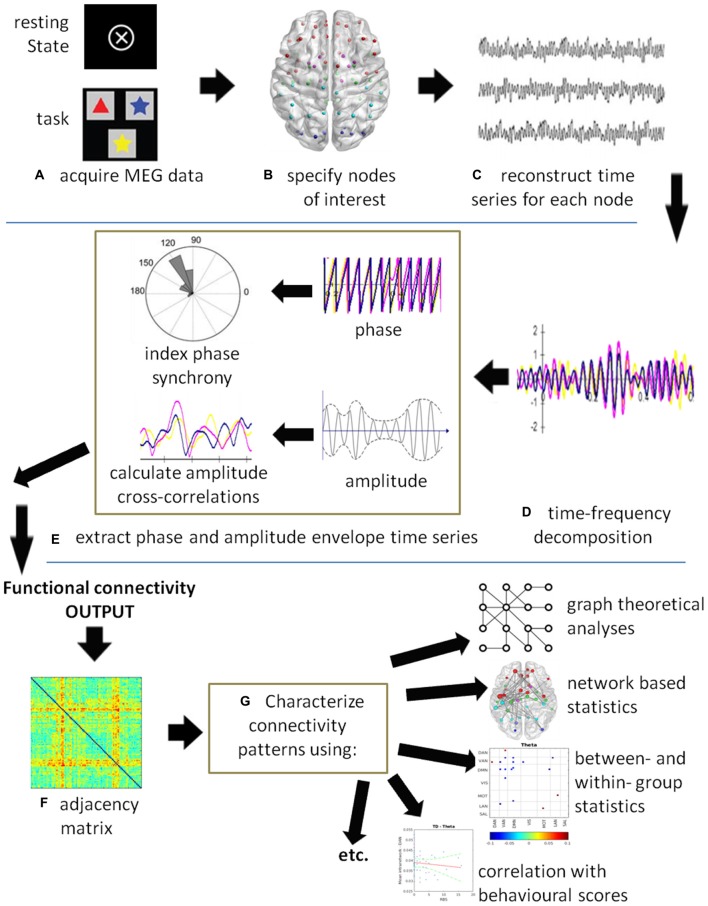
**A schematic of a generic magnetoencephalography (MEG) connectivity pipeline. (A)** MEG data can be acquired with or without a task. **(B)** Nodes of interest can be derived from specific coordinates obtained by source analysis, from the literature, or using either a grid- or atlas-based approach. **(C)** A time series is reconstructed for each node of interest. **(D)** Time-series decomposition is most commonly completed using a Hilbert or wavelet transform, although other methods can be used. At this stage, the data could be submitted to causality analysis to compute effective connectivity. **(E)** The phase and/or amplitude envelope information is extracted and correlations computed between all node pairs at each time point. Commonly used are the phase lag index (PLI), weighted PLI (wPLI), sometimes the phase locking value (PLV), and amplitude correlations. **(F)** The resultant output is an adjacency matrix showing connectivity between all node pairs. In this example, a color plot is used where red indicates highly connected nodes, although other types of plots may be used. **(G)** The connectivity results can be submitted to statistics depending on the question of interest. For example, graph theoretical metrics and network based statistics can be used to characterize the connectivity patterns in the networks. Group level statistics can be conducted using partial-least squares (PLS) or permutation testing. Individual scores on behavioral and neuropsychological assessments can be correlated with connectivity measures and submitted to a regression analysis.

Functional connectivity is defined as the “temporal correlations between remote neurophysiological events”, and effective connectivity is defined as “the influence [that] one neural system exerts over another” (Friston, 1994, p. 57). Thus, functional connectivity is a statistical measure that deals with the observation of correlated activity between remote brain areas under the assumption that areas behaving in a highly correlated manner must be working together. Functional connectivity does not provide insight into how these correlations are mediated and whether they are both essential to the process, or just correlated outputs driven by another process. Effective connectivity, on the other hand, deals with directions of influence and causality (Friston, 1994). Figure 1 illustrates a generic MEG connectivity pipeline from acquisition through connectivity computation to data visualization, characterization and statistic comparisons. The figure legend provides specifics on each of the steps.

**Figure 2:**
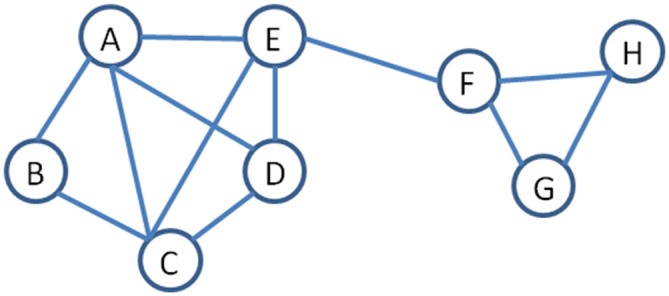
**An example of a network to illustrate terminology used in graph theory (Stam, 2004).** *Nodes* are the objects in the graph and are represented by a letter. Nodes are connected by *edges*, represented by the lines. *Path length* is the number of edges between two pairs of nodes, for example, the path length between B to H is 4. *Degree* is a measure of centrality and refers to the number of edges joining into a node, for example, A has a degree of 4 while H has a degree of 2. *Hub* is a measure of importance and nodes that are hubs have a high number of edges, for example, A and C are both hubs. *Neighborhood* refers to a set of adjacent nodes; thus, there are two neighborhoods in this example (A–E) and (F–H). The edge between E and F is referred to as a *bridge*, as it joins two neighborhoods. Node *strength* is another important concept and is a measures of the connectedness of a node’s neighbors to each other.

Because of the high temporal resolution of MEG, this technique can capture neuronal activity in the time domain as well as neuronal oscillatory activity in the time-frequency domain (for a review, see Pizzella et al., 2014). Thus, MEG connectivity can be computed based on correlated amplitude changes, which to some extent mimics fMRI functional connectivity (Brookes et al., 2011), and/or correlated oscillatory changes in the phase of the signals, which is a measure of synchronization and desynchronization (Schnitzler and Gross, 2005) between populations of neurons. This ability to quantify correlated amplitude and spectral changes between regions offers a perspective of the mechanisms and dynamics of brain connectivity that were not accessible without this level of temporal resolution. As well, the application of MEG functional connectivity patterns to graph theoretical analysis, organizes and visualizes the output into networks where important properties, such as node strength and path length can be identified (Stam, 2004). Figure 2 illustrates a graph or network and provides definitions for a number of common terms used in describing neural networks using graph theory terminology.

When initial studies suggested that brain functional connectivity did not overlay exactly onto brain structural connectivity via large fiber tracts, the question of which mechanisms underlie brain communication was brought to the forefront. The surprising answer came from the field of cellular electrophysiology and neuronal oscillations. It is known that neural populations can be coupled via chemical or electrical synapses, and once coupled, cell populations will engage in synchronized rhythmic activity. This oscillatory activity provides windows of alternating reduced and enhanced excitability that serve as a dynamic gating mechanism for the exchange of information. The dynamic gating of communication can occur between distributed nodes but have an overall organization based on a relatively stable structural connectome, and in fact, are organized in a hierarchical system that is responsible for the local-global integration of information whereby lower frequencies are involved in long-range global communication and higher frequencies in local interactions (von Stein and Sarnthein, 2000). At rest, neuronal populations have a preferred oscillatory frequency but when engaged in a task, the oscillatory dynamics of that structure will change and other oscillation frequencies will become dominant. These oscillations within distributed cell assemblies can co-occur, interact with and modulate each other thereby transmitting information over long distances (for a review, please see Buzsáki et al., 2013). Further, the disruption of these processes, at either the neurophysiological or neuroanatomical level, can lead to cognitive dysfunction and/or brain disease.

## MEG Studies of Functional Connectivity in Patients with Epilepsy

The most widespread clinical application of MEG has been in patients with localization-related epilepsy in whom surgical treatment of the epilepsy is being considered (see Bagić et al., 2011 for clinical guidelines for epilepsy recordings). Epilepsy surgery is the standard of care for appropriately selected adults and children with medically refractory epilepsy (Wiebe and Jetté, 2012). Surgical treatment for epilepsy is highly effective, has durable benefits, and can result in far better outcomes with respect to seizure freedom, improved quality of life, and reduction of the psychosocial comorbidities that accompany drug resistant epilepsy than continued medical treatment (Wiebe and Jetté, 2012; Jette et al., 2014).

A successful outcome from epilepsy surgery is generally defined as a seizure-free state with no imposition of neurological deficit (Snead, 2001). The key to this goal is to identify precisely the epileptogenic zone, which is defined as the area necessary and sufficient for the generation of habitual seizures, and the smallest amount of tissue that can be removed to achieve a seizure-free outcome (Obeid et al., 2009; Engel, 2013). In order to achieve seizure-freedom without an imposition of a neurologic deficit, one needs to lateralize and then localize the epileptogenic zone as well as localize eloquent function in the involved hemisphere. The use of MEG in the pre-surgical diagnostic evaluation of epilepsy candidacy in adults and children with medically refractory localization-related epilepsy contributes significantly to all three of these goals. Specifically, potential roles for MEG in epilepsy surgery include localization of the epileptogenic zone in conjunction with other non-invasive neurophysiological and imaging modalities (Lim et al., 2013; Jayakar et al., 2014), contribution to the decision making relevant to the indication for invasive monitoring and guidance of intracranial electrode placement as well as localization of eloquent function (Paetau and Mohamed, 2013). There are ample data from multiple centers that attest to the high sensitivity of MEG for epileptic activity and the importance of this modality in surgical decision-making (for example: Sutherling et al., 2008; Knowlton et al., 2009; Otsubo et al., 2010; Widjaja et al., 2013).

Despite the increasing and improved use of MEG, often in conjunction with other localization methods such as PET, SPECT and subdural grids, seizure control following epilepsy surgery even in carefully selected patients is not always optimal. In fact, it is estimated that epilepsy surgery only achieves complete and sustained seizure control in approximately 50% of patients with a focal neocortical epilepsy (Najm et al., 2013) and 75% with a mesial temporal lobe epilepsy (Spencer and Huh, 2008; Englot et al., 2013). These failures in surgical treatment are thought to stem from both an incomplete delineation, and thus resection, of the epileptogenic zone, as well as an incomplete understanding of the brain network in epilepsy (Englot et al., 2015). However, all that said, MEG does have a predictive value in epilepsy surgery. The complete resection of MEG clusters (defined as 6 or more spike sources with 1 cm or less between adjacent sources) has been shown to be correlated with post-surgical seizure freedom (Iida et al., 2005; Knowlton et al., 2009; Otsubo et al., 2010; Jung et al., 2013; Albert et al., 2014). Conversely, diffuse MEG spike sources indicate less likelihood for a localized seizure onset zone; therefore, this finding should weigh against invasive monitoring in the decision making process (Jung et al., 2013). Finally, it should be noted that MEG has been shown to be helpful in delineating an area of epileptogencity for subsequent resection in patients with an MRI-negative epilepsy (Rheims et al., 2013).

Some of the first MEG functional connectivity studies have reported abnormal increased connectivity in the theta-alpha range in patients with absence seizures (Chavez et al., 2010), increased connectivity in the beta range in patients with complex partial seizures (Madhavan et al., 2013), increased connectivity in the beta-gamma bands in focal cortical dysplasia (Jeong et al., 2014), increased connectivity in idiopathic generalized epilepsy (Elshahabi et al., 2015), and increases in functional connectivity between the default mode and medial temporal areas indicated the laterality of temporal lobe epilepsy (Hsiao et al., 2015). As well, in patients with epilepsy secondary to a brain tumor, increased connectivity was correlated with an increased numbers of seizures, and seizure vulnerability was related to a disorganized brain network topology (Douw et al., 2010). Clearly, epileptic cortex is associated with aberrant network connectivity within and between brain regions although the exact nature of this relation is not yet known.

It has been suggested that increased connectivity across small distances, that is, aberrant local connectivity amongst a small group of neurons, might be indicative of an irregularity in neuronal excitability (Laufs, 2012) and may thus be an important factor in epileptiform spike generation. For this reason, mapping the connectivity pattern around seizure onset zones might provide information about how seizures propagate and the extent of the epileptic network. At this time, these ideas have only been explored with electrocorticography (ECoG) and high-density EEG data. For example, one study measured effective connectivity from ECoG to discriminate connectivity associated with epileptic vs. eloquent cortex (Asano et al., 2013), while another used changes in patterns of functional connectivity to predict upcoming seizures and localize the seizure onset zone (van Mierlo et al., 2014). A study using high-density EEG found that neuropsychological deficits were related to different patterns of connectivity dysfunctions in right vs. left temporal lobe epilepsies (Coito et al., 2015). These ideas have not yet been applied to MEG although these electrophysiological studies suggest that these methods would be effective in MEG and would allow this information to be gathered non-invasively. However, MEG connectivity analysis of atypical expressive language laterality has been shown to be associated with the alteration of large-scale network integration in children with medically-refractory localization-related epilepsy (Ibrahim et al., 2015). Similarly MEG analysis has shown an association between network perturbations and neurocognitive outcome in children with medically refractory epilepsy (Ibrahim et al., 2014a,b).

MEG functional connectivity studies have not yet been applied to pre-surgical functional mapping protocols, although studies in controls are being reported and will lay the foundation for clinical studies. In a group of control adults performing a verb generation task, left hemisphere language networks were identified that included canonical and extra-canonical language areas which interacted through short-range synchronization in the gamma band with long range modulation in the theta band (Doesburg et al., 2012). Effective connectivity measurements in a group of typically developing school-aged children confirmed posterior-to-anterior flow of information, from visual fusiform areas to language areas in the supramarginal and angular gyri to inferior frontal areas (Simos et al., 2013) with measures of effective connectivity correlating with age (Kadis et al., 2016). Further, left hemisphere connectivity in the theta band correlated with performance on receptive language tasks in pre-school children (Kikuchi et al., 2011) while task-dependent theta-band synchronization for expressive language increased with age through adolescence and correlated with neuropsychological assessments of language ability (Doesburg et al., 2016). Finally, a study has demonstrated concordance between MEG and fMRI measures of functional connectivity on a naming task, with MEG providing additional information from different frequency bands (Liljeström et al., 2015b) that support specific functional roles for the different frequency bands with beta frequencies playing a more facilitatory role and gamma oscillatory synchronization playing a more inhibitory role in speech production (Liljeström et al., 2015a).

With these first studies of MEG language-related functional connectivity in control adults and typically developing children, the stage is set for translation of these methods into the clinical realm to examine the impact of disease on network changes. The patterns of network changes will inform us as to how the injured brain has re-organized itself or compensated so as to preserve function.

## Function Beyond Structure: The Value of Adding MEG Studies to Functional Neurosurgery

Hart et al. (2016) have provided a comprehensive overview of the relevance of graph theoretical analysis and functional connectivity computations to neurosurgical practice. The authors emphasize the current paradigm shift where brain function is considered a product of information exchange between members of a neural network, and functional mapping includes a description of the network properties.

It follows that abnormalities in brain connectivity may be responsible for a number of brain dysfunctions. In situations where there are known structural abnormalities, for example, a brain tumor, there is a non-specific but observable loss of functional connectivity (Bartolomei et al., 2006; Guggisberg et al., 2008) which could be related to a decrease in neurocognitive function (Bosma et al., 2008). A recent study used resting state MEG connectivity analyses to assess the impact of tumors in eloquent areas (Martino et al., 2011) concluded that these methods held promise as a potential avenue for non-invasive pre-surgical planning. In fact, there are now a number of studies examining MEG functional connectivity changes in a variety of neurological and psychiatric conditions (e.g., Stam, 2010; Dunkley, 2015; Pang et al., 2016). The data accumulating from these studies increasingly illustrate the value of exploring functional network abnormalities that are not observable at the level of structural connectivity. That is, there are an increasing numbers of examples where the brain’s gross anatomy and structure are normal on MRI but investigations at the brain’s microstructural level demonstrate alterations that impair neuronal output and connectivity with a resultant impairment of function.

While most clinical studies show connectivity differences at the group level, recent new approaches allow the correlation of network metrics with individual neuropsychological and/or behavioral scores. For example, in a study from our lab, soldiers with post-traumatic stress disorder were found to have significantly higher connectivity in the right parietal cortex, and the extent of this hyper-connectivity was directly correlated with their clinical symptoms of depression and anxiety (Dunkley et al., 2015). This is one recent example which demonstrates the possibility of extracting network metrics at an “*n* = 1” level to allow correlation and interpretation for a single patient.

While far from being ready to take this approach into the operating room, it is a curious mind and the persistent movement forward in small steps that culminates in a large leap of knowledge. The journal *Neurosurgery* published a special article entitled “Mapping the Human Connectome” (Toga et al., 2012) which summarizes, “For the foreseeable future, a comprehensive description of the complete connectome of even a single human brain might be viewed as unattainable. But the science of connectomics is devoted to filling in the gaps”. We think, at this stage, neurosurgeons can play a significant role in “filling in the gaps” to test the multitude of functional connectivity hypotheses and findings that are filling the literature. The classic lesion studies by Broca, or stimulation studies by Penfield, were instrumental in developing the localization models of function. Like these pioneers, modern day neurosurgeons can parlay their observations of brain disease to answer questions regarding the characteristics, integrity and plasticity of functional neural networks and brain dynamics. At that time, we hope that MEG will be included in the repertoire of neuroimaging studies. MEG, with its high resolution in the temporal and oscillatory domains, is ideally suited to explore the oscillatory dynamics that underlie brain functional communication and complements the information obtained by other neuroimaging modalities.

With regards to functional neurosurgery, the increasing evidence that data acquired from MEG can model neural networks with high fidelity raises the possibility of simulating the impact of a resection and predicting functional outcome. For example, using MEG data collected from a patient with epilepsy, connectivity algorithms would identify the hubs and connections within the epilepsy and functional (i.e., language or motor) networks for that individual. With this information, a model of these networks could be created and subjected to simulated resections. A simulated resection may involve removal of a putative epileptogenic zone that impinges on functional cortex. The model could then predict whether the core epileptogenic zone was identified and whether sufficient disconnections were made to preclude seizure propagation. At the same time, the model could predict whether key hubs, or compensatory hubs, with sufficient connections were retained within the functional network to preserve core behaviors. Various simulated resection margins could be tested until the right balance of disease removal and functional sparing was achieved. While MEG studies of functional connectivity are not ready for this level of translation, the ability of MEG to record true neurophysiological activity with high fidelity brings the possibility of neural network modeling and simulations into the realm of possibility. This potential advancement raises exciting possibilities for future applications and approaches in functional neurosurgery.

## Summary

With access to new neuroimaging tools and methods for understanding brain function, the field of neurosurgery has an opportunity to grow in its ability to offer more precise surgical margins that approach disease more aggressively while better preserving functional networks. The measurement of structural and functional connectivity will open a window on brain function, communication and organization that will offer both theoretical and practical insights. The addition of MEG studies, with its high resolution in the time, space and oscillatory domain, will allow a look into brain communication that augments and fine tunes what can be learned from brain structure.

Within the field of neuroscience, a multi-layered paradigm shift has been occurring. First, our traditional segregationist view of function, while adequate for identifying “hubs” of activity, is woefully inadequate for describing the complexity of function. Rather, the introduction of metrics which describe the properties of both the hubs and connections seem to provide a more accurate picture of how function is subsumed in the brain. Second, our traditional view of a “resting” brain is now thought to be incorrect. In his recent review of this topic, Raichle (2015) uses the term “restless” brain, reflecting the shift from considering non-task-related activity as “noise”, to a new understanding that the brain is always working and there are functional brain networks active during non-task periods. Finally, it is now understood that these intrinsic functional brain networks are organized and can be identified by high inter-regional correlations in phase or amplitude. Network communication occurs by modulation and integration of these inter- and intra-regional oscillations. The MEG is well poised for exploring questions of intrinsic brain networks and neuronal oscillations.

The application of computational neuroscience, and the identification of functional connections will improve our understanding of how distinct brain regions interact, and whether a region is essential or collateral to a function, Understanding the role that a particular region and its network connections play in a function will be very helpful in neurosurgical decision making, especially in a diseased brain, where developmental and neuroplastic processes may have shifted aspects of a function to another region. While the field of computational neuroscience is still very young, it already holds great promise for what it may offer to the field of neurosurgery for pre-surgical planning and the improvement of neurosurgical care.

## Author Contributions

EWP and OCS contributed equally to the writing of this article.

## Conflict of Interest Statement

The authors declare that the research was conducted in the absence of any commercial or financial relationships that could be construed as a potential conflict of interest. The handling Editor declared a shared affiliation, though no other collaboration, with the authors and states that the process nevertheless met the standards of a fair and objective review.
